# Supplementary material for: The substrate specificity of the human TRAPPII complex’s Rab-guanine nucleotide exchange factor activity
Source: Commun Biol. 2020 Dec 4;3:735. doi: 10.1038/s42003-020-01459-2 (PMC7719173; doi:10.1038/s42003-020-01459-2)
Supplement: Supplementary file 1 — Supplementary Information [file 42003_2020_1459_MOESM1_ESM.pdf]

Supplementary Information

The substrate specificity of the human TRAPPII complex's Rab-guanine nucleotide exchange factor activity

Meredith L Jenkins[1], Noah J Harris[1*], Udit Dalwadi[2*], Kaelin D Fleming[1], Daniel S. Ziemianowicz[3], Atefeh Rafiei[4], Emily M Martin[1], David C Schriemer[3,4], Calvin K Yip[2], John E Burke[1,2%]

[1]Department of Biochemistry and Microbiology, University of Victoria, Victoria, British Columbia, Canada V8W 2Y2

[2]Department of Biochemistry and Molecular Biology, University of British Columbia, Vancouver, British Columbia, Canada V6T 1Z3

[3]Department of Biochemistry and Molecular Biology, University of Calgary, Calgary, Alberta, Canada T2N 4N1

[4]Department of Chemistry, University of Calgary, Calgary, Alberta, Canada T2N 4N1

* These authors contributed equally

[%]To whom correspondence should be addressed: John E. Burke

Tel: 1-250-721-8732, email: jeburke@uvic.ca

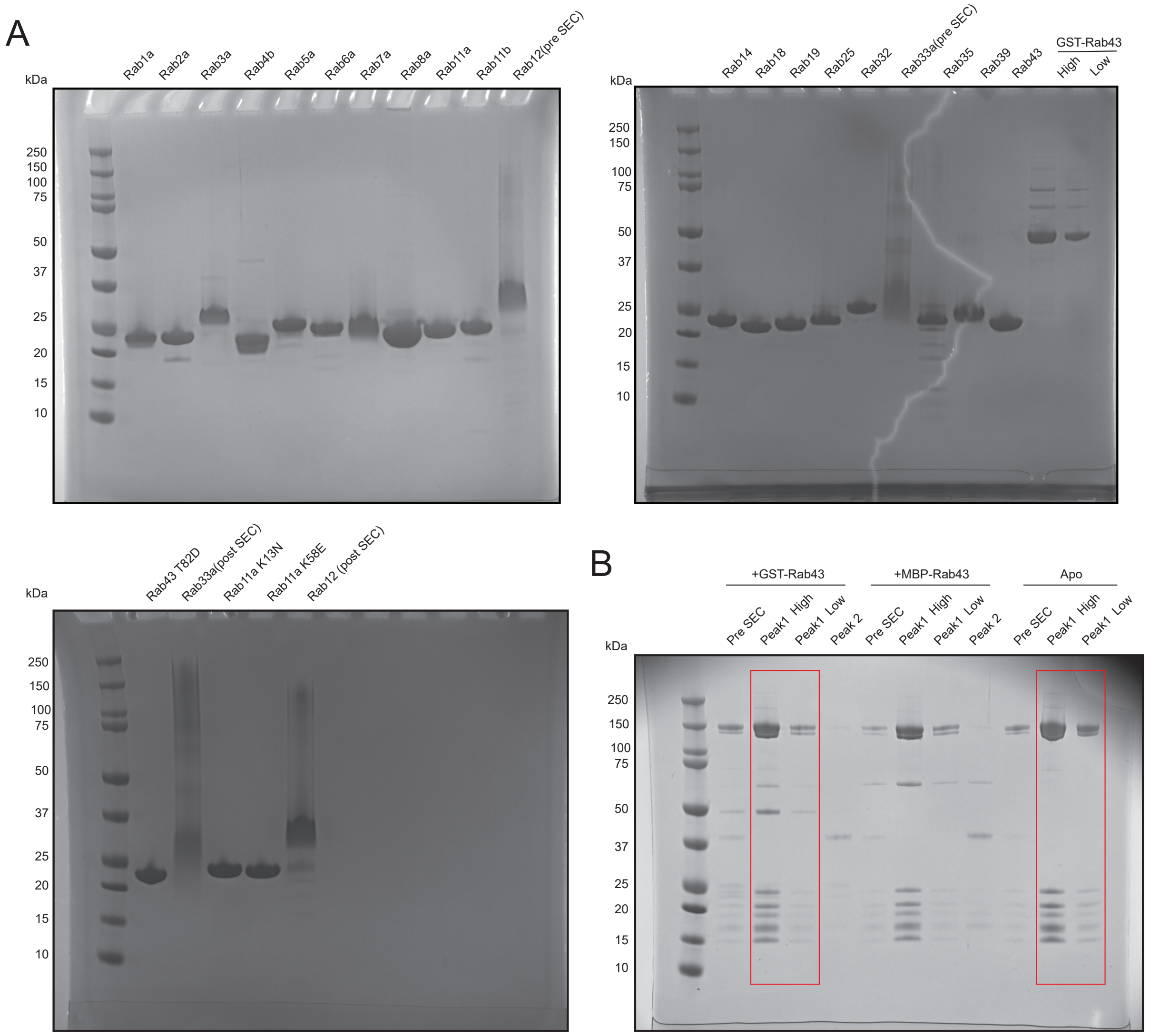

Supplemental Figure 1. SDS-PAGE gel of purified proteins.
(A) All Rab GTPases used in the study are shown on the gels (5ug loaded). Rab33a did not produce a clear band, so MS/MS was utilized to confirm its purification (100% coverage). High and low labels refer to protein amount loaded (High=5ug, Low=2.5ug) (4-12% NuPAGE gradient gel run at 200V for 45 min and stained with Coomassie Brilliant Blue dye).
(B) Uncropped gel of TRAPPII used in figures 1 and 3. Lanes used in the figures are boxed. Peak 1 refers to the first SEC peak on Fig 3a, and peak2 refers to the second peak on Fig 3a. High and low labels refer to protein amount loaded (High=4ug, Low=0.67ug) (4-12% NuPAGE gradient gel run at 200V for 45 min and stained with Coomassie Brilliant Blue dye).

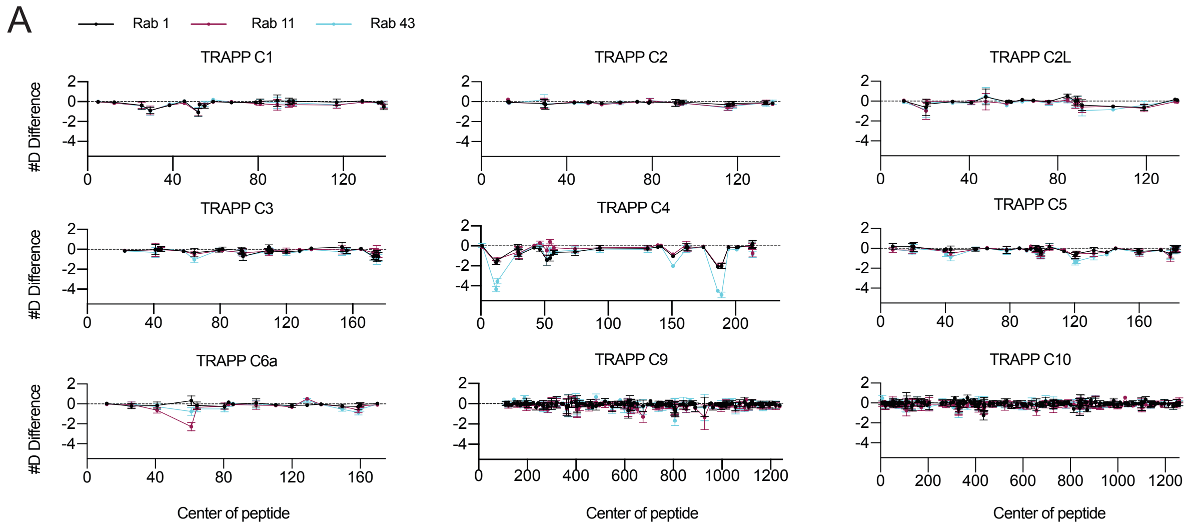

Supplemental Figure 2. Raw differential HDX-MS analysis of TRAPPII bound to Rab isoforms (refers to Figure 3).

(A) The number of deuteron difference for peptides analysed over the entire deuterium exchange time course for Rab1, Rab11 or Rab43 in the presence of TRAPPII subunit. Every point represents the central residue of an individual peptide. Number of deuteron differences from Apo to complex for Rab43 are depicted in cyan, Rab11 in magenta, and Rab1 in blue. The full deuterium exchange data is also available as Source data.

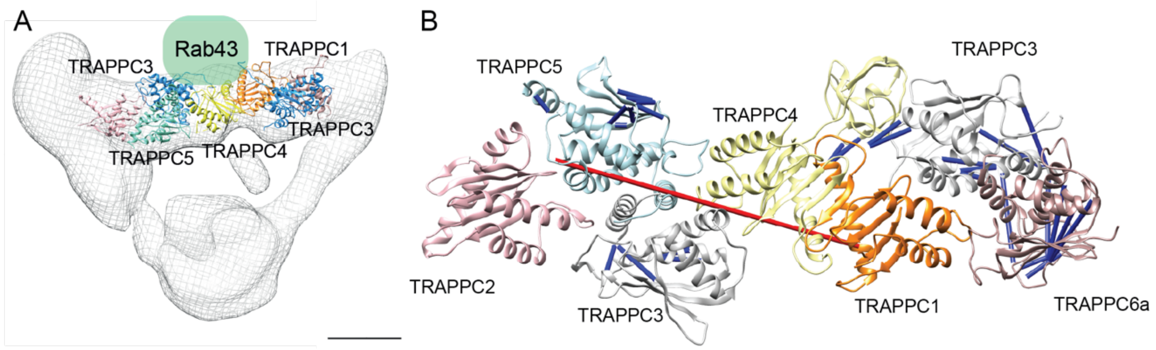

Supplemental Figure 3. Analysis of EM reconstruction and chemical crosslinks in the TRAPP core model (refers to Figure 4).

(A) EM density maps of TRAPPII fitted with the structure model for the human TRAPP core with a modelled position of Rab43. Scale bar is 50 Å. The exact orientation of the TRAPP core is ambiguous, and could also be in an alternate conformation rotated 180 degrees.

(B) Identified crosslinks that fit within the distance constraints of the LC-SDA and SDA crosslinkers are shown in blue (26 in total), with a single outlier between TRAPPC5 and TRAPPC1 that does not fit within the distance restraints shown in red. The maximum allowed distance ($C\alpha$-$C\alpha$) was set as 30Å for SDA and 35Å for LC-SDA.

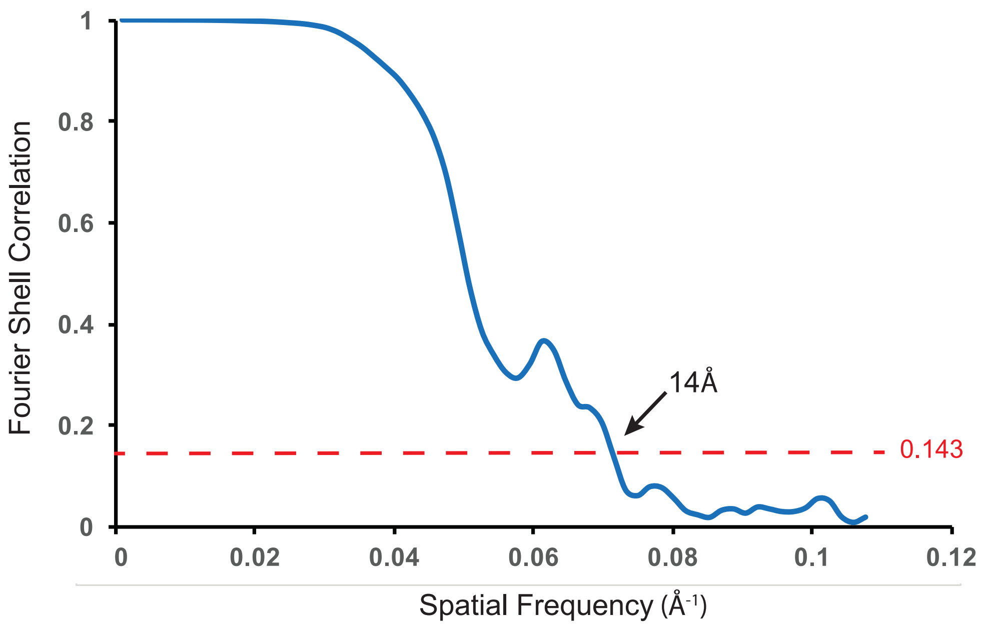

Supplemental Figure 4. Gold-standard Fourier shell correlation curve showing the resolution of the TRAPPII model.

Supplemental table 1. All plasmids used in this study.

Name	Plasmid	Protein(s)	Sequence(s)	Modifications/Tags	Source
MJ153<br><br>[TRAPPIIa]	pBIG1a	TRAPPC1;<br>TRAPPC2L;<br>TRAPPC5;<br>TRAPPC6a;<br>TRAPPC10;	1-145;<br>1-140;<br>1-188;<br>1-173;<br>1-1259	TRAPPC10 – C-term His Tag (TEV)	This Paper
MJ85<br><br>[TRAPPIIb]	pBIG1a	TRAPPC3;<br>TRAPPC2;<br>TRAPPC4;<br>TRAPPC9	1-180;<br>1-140;<br>1-235;<br>1-1148	TRAPPC3 – C-term Strep Tag (TEV)	This Paper
MJ117	pOPTGcH	Rab1	1-203	N-term GST Tag(TEV); C-term His Tag	This Paper
MJ37	pOPTGcH	Rab2a	1-210	N-term GST Tag(TEV); C-term His Tag	Jenkins *et al.,* 2018
EM11	pOPTGcH	Rab3a	1-217	N-term GST Tag(TEV); C-term His Tag	This Paper
MJ36	pOPTGcH	Rab4b	1-210	N-term GST Tag(TEV); C-term His Tag	Jenkins *et al.,* 2018
EM13	pOPTGcH	Rab6a	1-205	N-term GST Tag(TEV); C-term His Tag	This Paper
NH20	pOPTGcH	Rab7a	1-204	N-term GST Tag(TEV); C-term His Tag	This Paper
MJ40	pOPTGcH	Rab8a	1-203	N-term GST Tag(TEV); C-term His Tag	Jenkins *et al.,* 2018
EO7	pOPTGcH	Rab11a	1-211	N-term GST Tag(TEV); C-term His Tag	Jenkins *et al.,* 2018
JM238	pOPTGcH	Rab11a K13N	1-211	K13N<br>N-term GST Tag(TEV); C-term His Tag	Jenkins *et al.,* 2018
MJ119	pOPTGcH	Rab11a K58E	1-211	K58E<br>N-term GST Tag(TEV); C-term His Tag	This Paper
MJ28	pOPTGcH	Rab11b	1-213	N-term GST Tag(TEV); C-term His Tag	Jenkins *et al.,* 2018
MJ39	pOPTGcH	Rab12	1-242	N-term GST Tag(TEV); C-term His Tag	Jenkins *et al.,* 2018
MJ38	pOPTGcH	Rab14	1-212	N-term GST Tag(TEV); C-term His Tag	Jenkins *et al.,* 2018
EM15	pOPTGcH	Rab18	1-198	N-term GST Tag(TEV); C-term His Tag	This Paper
MJ167	pOPTGcH	Rab19	1-214	N-term GST Tag(TEV); C-term His Tag	This Paper
MJ27	pOPTGcH	Rab25	1-208	N-term GST Tag(TEV); C-term His Tag	Jenkins *et al.,* 2018
NH19	pOPTGcH	Rab29	1-201	N-term GST Tag(TEV); C-term His Tag	This Paper
NH21	pOPTGcH	Rab32	1-223	N-term GST Tag(TEV); C-term His Tag	This Paper
EM17	pOPTGcH	Rab33a	1-234	N-term GST Tag(TEV); C-term His Tag	This Paper
Em19	pOPTGcH	Rab35	1-199	N-term GST Tag(TEV); C-term His Tag	This Paper
EM23	pOPTGcH	Rab39a	1-214	N-term GST Tag(TEV); C-term His Tag	This Paper
EM25	pOPTGcH	Rab43	1-209	N-term GST Tag(TEV); C-term His Tag	This Paper
MJ161	pOPTGcH	Rab43 T82D	1-209	T82D<br>N-term GST Tag(TEV); C-term His Tag	This Paper

Supplemental Table 2. HDX-MS statistics for Figure 3.

Data set	TRAPPII C1	TRAPPII C2	TRAPPII C2L	TRAPPII C3
HDX reaction details	%D_2O=84.8%<br>$pH_{(read)}$=7.5<br>Temp=18ºC	%D_2O=84.8%<br>$pH_{(read)}$=7.5<br>Temp=18ºC	%D_2O=84.8%<br>$pH_{(read)}$=7.5<br>Temp=18ºC	%D_2O=84.8%<br>$pH_{(read)}$=7.5<br>Temp=18ºC
HDX time course (seconds)	3, 30, 300, 3000	3, 30, 300, 3000	3, 30, 300, 3000	3, 30, 300, 3000
HDX controls	N/A	N/A	N/A	N/A
Back-exchange	Corrected based on %D_2O	Corrected based on %D_2O	Corrected based on %D_2O	Corrected based on %D_2O
Number of peptides	21	22	20	27
Sequence coverage	96.6%	94.3%	92.1%	83.5%
Average peptide /redundancy	Length=13.2<br>Redundancy= 1.9	Length=13.0<br>Redundancy= 2.0	Length=10.9<br>Redundancy= 1.6	Length=13.6<br>Redundancy= 2.0
Replicates	3 (2 for Rab43 3s and 2 for Rab11 300s)	3 (2 for Rab43 3s and 2 for Rab11 300s)	3 (2 for Rab43 3s and 2 for Rab11 300s)	3 (2 for Rab43 3s and 2 for Rab11 300s)
Repeatability	Average StDev=0.5%	Average StDev=0.4%	Average StDev=0.7%	Average StDev=0.6%
Significant differences in HDX	>4% and >0.4 Da and unpaired t-test ≤0.01	>4% and >0.4 Da and unpaired t-test ≤0.01	>4% and >0.4 Da and unpaired t-test ≤0.01	>4% and >0.4 Da and unpaired t-test ≤0.01

TRAPPII C4	TRAPPII C5	TRAPPII C6a	TRAPPII C9	TRAPPII C10
%D_2O=84.8%<br>$pH_{(read)}$=7.5<br>Temp=18ºC	%D_2O=84.8%<br>$pH_{(read)}$=7.5<br>Temp=18ºC	%D_2O=84.8%<br>$pH_{(read)}$=7.5<br>Temp=18ºC	%D_2O=84.8%<br>$pH_{(read)}$=7.5<br>Temp=18ºC	%D_2O=84.8%<br>$pH_{(read)}$=7.5<br>Temp=18ºC
3, 30, 300, 3000	3, 30, 300, 3000	3, 30, 300, 3000	3, 30, 300, 3000	3, 30, 300, 3000
N/A	N/A	N/A	N/A	N/A
Corrected based on %D_2O	Corrected based on %D_2O	Corrected based on %D_2O	Corrected based on %D_2O	Corrected based on %D_2O
27	36	37	141	161
95.9%	92.6%	88.4%	85.8%	91.9%
Length=13.1<br>Redundancy= 1.5	Length=13.2<br>Redundancy= 2.5	Length=11.9<br>Redundancy= 1.2	Length=13.6<br>Redundancy= 1.5	Length=13.6<br>Redundancy= 1.7
3 (2 for Rab43 3s and 2 for Rab11 300s)	3 (2 for Rab43 3s and 2 for Rab11 300s)	3 (2 for Rab43 3s and 2 for Rab11 300s)	3 (2 for Rab43 3s and 2 for Rab11 300s)	3 (2 for Rab43 3s and 2 for Rab11 300s)
Average StDev=0.6%	Average StDev=0.6%	Average StDev=0.4%	Average StDev=0.6%	Average StDev=0.6%
>4% and >0.4 Da and unpaired t-test ≤0.01	>4% and >0.4 Da and unpaired t-test ≤0.01	>4% and >0.4 Da and unpaired t-test ≤0.01	>4% and >0.4 Da and unpaired t-test ≤0.01	>4% and >0.4 Da and unpaired t-test ≤0.01
