# Supplementary material for: The substrate specificity of the human TRAPPII complex’s Rab-guanine nucleotide exchange factor activity
Source: Commun Biol. 2020 Dec 4;3:735. doi: 10.1038/s42003-020-01459-2 (PMC7719173; doi:10.1038/s42003-020-01459-2)
Supplement: Supplementary file 3 — Description of Additional Supplementary Files [file 42003_2020_1459_MOESM3_ESM.pdf]

Description of Additional Supplementary Files

File Name: Supplementary Data 1
Description: **Summary of all HDX-MS peptide data (refers to Fig. 3**). The charge state (Z), residue start (S), residue end number (E), and retention time (RT) are displayed for every peptide. Data listed is the mean of 3 independent experiments, with SDs presented. Time points are labeled, and the relative level of HDX is colored according to the legend.

File Name: Supplementary Data 2
Description: **All raw data used in main text figures.** Data used to generate each figure panel is shown on different tabs
